# Strengthening capacity for pandemic preparedness among health care professionals through interdisciplinary online learning: a participatory mixed-methods evaluation

**DOI:** 10.3389/fpubh.2026.1879989

**Published:** 2026-08-26

**Authors:** Maryirene Ibeto, Imtiaz Mahmood Tahir, Mukhtar Danmallam, Beatrice Kamau, Victor Mtaki, Paul Msanzya Zulu, Fred Tusabe, Helen Wangai, Chimwemwe Waya, Elizabeth Clery, Chizaram Fide-Nwaogu, Joanna Wild, Farhana Haque, Femi Nzegwu

**Affiliations:** 1 London School of Hygiene and Tropical Medicine (LSHTM), London, United Kingdom; 2 College of Allied Health Professionals, Government College University, Faisalabad, Pakistan; 3 Department of Community Medicine, Ahmadu Bello University Teaching Hospital, Zaria, Nigeria; 4 County Health, Nairobi, Kenya; 5 International Training and Education Centre for Health (I-TECH), Tanzania Department of Global Health, University of Washington, Seattle, WA, United States; 6 Zambia National Public Health Institute, Zambia National Public Health Institute (ZNPHI), Lusaka, Zambia; 7 Gillings School of Global Public Health University of North Carolina at Chapel Hill, Chapel Hill, NC, United States; 8 USAID Medicines Technologies and Pharmaceutical Services (MTaPS) Program, Nairobi, Kenya; 9 World Health Organization, Lilongwe, Malawi; 10 Health Emergency Preparedness and Response Department. Nigeria Centre for Disease Control and Prevention (NCDC), Abuja, Nigeria; 11 OL4ALL, Oxford, United Kingdom; 12 UK Public Health Rapid Support Team (UK-PHRST), Department of Infectious Disease Epidemiology and Dynamics, London School of Hygiene and Tropical Medicine (LSHTM), London, United Kingdom

**Keywords:** capacity strengthening, interdsiciplinary, pandemic preparedness, public health education, workforce development

## Abstract

**Introduction:**

Between January and March 2022, the UK Public Health Rapid Support Team delivered a master’s-level short course, Pandemics: Emergence, Spread and Response, designed to strengthen interdisciplinary knowledge and skills for pandemic preparedness. This study evaluated the course’s perceived usefulness, outcomes, and longer-term impacts on professional practice, one year after completion.

**Methods:**

A participatory convergent mixed-methods design was used, involving course alumni as co-evaluators. Quantitative data were collected through an online survey (n = 31; 62% response rate), while qualitative data were generated through focus group discussions (FGDs), value creation stories (VCS), and iterative workshops. Wenger, Trayner and de Laat’s Value Creation Framework informed the evaluation design and interpretation of findings. Quantitative analyses were exploratory and intended to identify patterns rather than test formal hypothesis.

**Results:**

Participants consistently reported that the course was relevant and useful to their professional roles. Exploratory analyses identified associations between perceived course usefulness and engagement, course content, delivery mode, learning platform, and facilitator diversity; these findings should be interpreted cautiously given the small sample size and exploratory design and should not be interpreted as causal. Qualitative findings provided explanatory depth by demonstrating how interactive pedagogy, interdisciplinary learning, practical case-based teaching, and exposure to diverse perspectives facilitated knowledge transfer and application in professional practice, generating value across Wenger’s cycles of immediate, potential, applied, realised and reframing value.

**Discussion:**

Overall, the findings suggest that interdisciplinary, learner-centred online training may support professional practice change when engagement, inclusivity, and contextual relevance are prioritised. This study demonstrates the value of participatory, mixed-methods evaluation and Wenger’s Value Creation Framework for understanding long-term learning outcomes in pandemic preparedness training.

## 1 Introduction

The COVID-19 pandemic underscored the urgent need for skilled public health professionals capable of preparing for, responding to, and mitigating the impact of emerging infectious diseases. Effective pandemic response requires interdisciplinary knowledge, cross-sectoral collaboration, and adaptive leadership across health systems and communities (1, 2). In recognition of these needs, the UK Public Health Rapid Support Team (UK-PHRST)—a partnership between the London School of Hygiene and Tropical Medicine (LSHTM) and the UK Health Security Agency (UKHSA)—developed a master’s-level short course titled *Pandemics: Emergence, Spread and Response* (3). The UK-PHRST supports rapid outbreak response, operational research and capacity strengthening in low- and middle-income countries (LMICs) (4, 5).

Between January and March 2022, the course was delivered to a cohort of public health professionals. The aim of the course was to develop participants’ knowledge and skills related to pandemic drivers, multi-disciplinary actors and response measures, while strengthening their ability to critically appraise and synthesise evidence for pandemic preparedness and response (3). Course developers adopted constructivist and social constructivist pedagogical approaches to support interdisciplinary learning (6). Interdisciplinary learning enables students to draw on knowledge and methods from multiple disciplines and integrate them to address complex problems that cannot be adequately addressed through single disciplinary approaches. Effective pandemic preparedness and response depend on coordinated action across multiple disciplines, sectors, and institutions, including public health, clinical medicine, epidemiology, laboratory sciences, social sciences, policy, and emergency management. Consequently, training programmes must equip professionals not only with technical expertise but also with the ability to understand, communicate, and collaborate across disciplinary boundaries. Interdisciplinary learning provides a mechanism for developing these competencies by exposing learners to diverse perspectives and enabling them to integrate knowledge from multiple fields when addressing complex public health challenges (7–10).

This part-time course used student-centred learning approaches, including group discussions and presentations, simulations, critical appraisal of the literature, debates with integrated feedback and assessments to support students in using higher-order analytical and reflective skills to critique, create, and develop their thinking—independently or with peers—on the topics under consideration. It adopted a blended instructional design combining asynchronous (self-directed) and synchronous (directed) learning over a 10-week period. Each week consisted of 20 learning hours, including 5 h of synchronous and 15 h of self-directed learning. The intended learning outcomes were to enable critical reflection, synthesis and application of knowledge in real-world pandemic preparedness and response contexts. The course was structured into five sequential modules delivered over 10 weeks, covering the continuum of pandemic preparedness and response. Topics included the drivers and history of emerging infectious diseases, pandemic prevention and global governance, surveillance and outbreak detection, multisectoral and One Health approaches to response, and strategies for strengthening future pandemic preparedness through collaboration, innovation, resilience, and equity (Additional information is included within the Supplementary Table S1). Assessment comprised both formative and summative components. Formative assessment was integrated throughout the course using weekly self-assessment quizzes, reflective writing exercises with model answers, and small-group activities that provided opportunities for tutor and peer feedback to support ongoing learning. The summative assessment consisted of an individual written assignment submitted at the end of the course, requiring participants to synthesise and apply learning from across the programme, drawing on their portfolio of reflective writing. Assignments were independently double marked using a pre-defined marking rubric aligned with the course learning outcomes.

The course attracted 50 participants from 20 countries, from Africa (39), Europe (3), East & South Asia (3), North America (2), South America (2), and Australia (1); and was delivered online over 10 weeks. Participants represented diverse disciplines including medicine, epidemiology, pharmacy, infectious disease control and water and sanitation.

Although a growing body of literature describes the expansion of online and blended learning for pandemic preparedness and global health education, most evaluations remain limited to short-term educational outcomes such as learner satisfaction, self-reported knowledge gain, or immediate competency development (11–14). Few studies examine whether and how such training translates into sustained changes in professional practice, institutional processes, or broader health system preparedness over time (15, 16). To our knowledge, most evaluations of training programmes rely primarily on researcher-defined outcome measures, with relatively few adopting participatory approaches that actively involve learners in defining and assessing value, outcomes, and impact in their own professional contexts (17, 18). As a result, there is limited empirical evidence on how interdisciplinary, online pandemic preparedness training contributes to longer-term capacity strengthening, particularly among globally diverse public health professionals.

While evaluations of professional education programmes frequently assess participant satisfaction, perceived learning, or knowledge acquisition, fewer studies examine how learning generates value beyond the classroom and contributes to changes in professional practice, organisational processes and wider systems. Wenger, Trayner and de Laat’s Value Creation Framework provides a useful lens for understanding these pathways by conceptualising learning as a process of value creation that progresses through interconnected cycles of immediate, potential, applied, realised and reframing value. The framework has been used to assess how learning influences individuals, organisations and networks over time, making it particularly relevant for evaluating complex capacity-strengthening and professional development interventions (19, 20).

One year after course completion, a participatory evaluation was conducted to examine the course’s usefulness (defined as participants’ assessment of the extent to which the course enhanced their professional knowledge, skills and ability to apply learning to pandemic preparedness and response in their workplace), the outcomes (the direct results of the learning and what changed as a result of the course) and broader impacts on participants’ professional practice, institutional policies and processes, and broader health system preparedness. The specific research question was “what value did participants derive from the *Pandemics: Emergence, Spread and Response* course, and how was this translated into professional, organisational and wider public health preparedness outcomes?” Unlike most evaluations of short courses, this evaluation was designed to capture longer-term professional and institutional effects and to explore mechanisms of learning transfer using both quantitative and qualitative approaches.

### 1.1 Contribution to policy and practice

This study contributes to the evidence base on public health education and pandemic preparedness in several important ways. First, it provides longitudinal evidence on how professional training may translate into practice and institutional processes beyond immediate post-course outcomes, offering insights into sustained workforce capacity strengthening. Second, it identifies key design features associated with learning transfer, including learner engagement, interdisciplinary content, and exposure to diverse perspectives, thereby providing empirically grounded insights relevant to the design of future capacity-strengthening initiatives. Third, the findings highlight implications for workforce development at the systems level, illustrating how training may contribute to policy processes, outbreak response roles, and organisational practices. Fourth, the study draws attention to equity and inclusion considerations in global health training, including accessibility challenges and the representation of perspectives from LMICs. Fifth, it demonstrates a transferable evaluation approach, integrating participatory and mixed methods designs to capture outcomes beyond immediate learning, including perceived changes in professional practice and institutional influence. Finally, it demonstrates the value of Wenger’s Value Creation Framework as a theory-informed approach for evaluating educational and capacity strengthening interventions. By tracing pathways from learning experiences to changes in practice, organisational improvement and systems-level influence, the framework provides a richer understanding of how professional education creates value over time and across multiple levels of impact.

## 2 Methods

### 2.1 Study design

**Figure 1:**
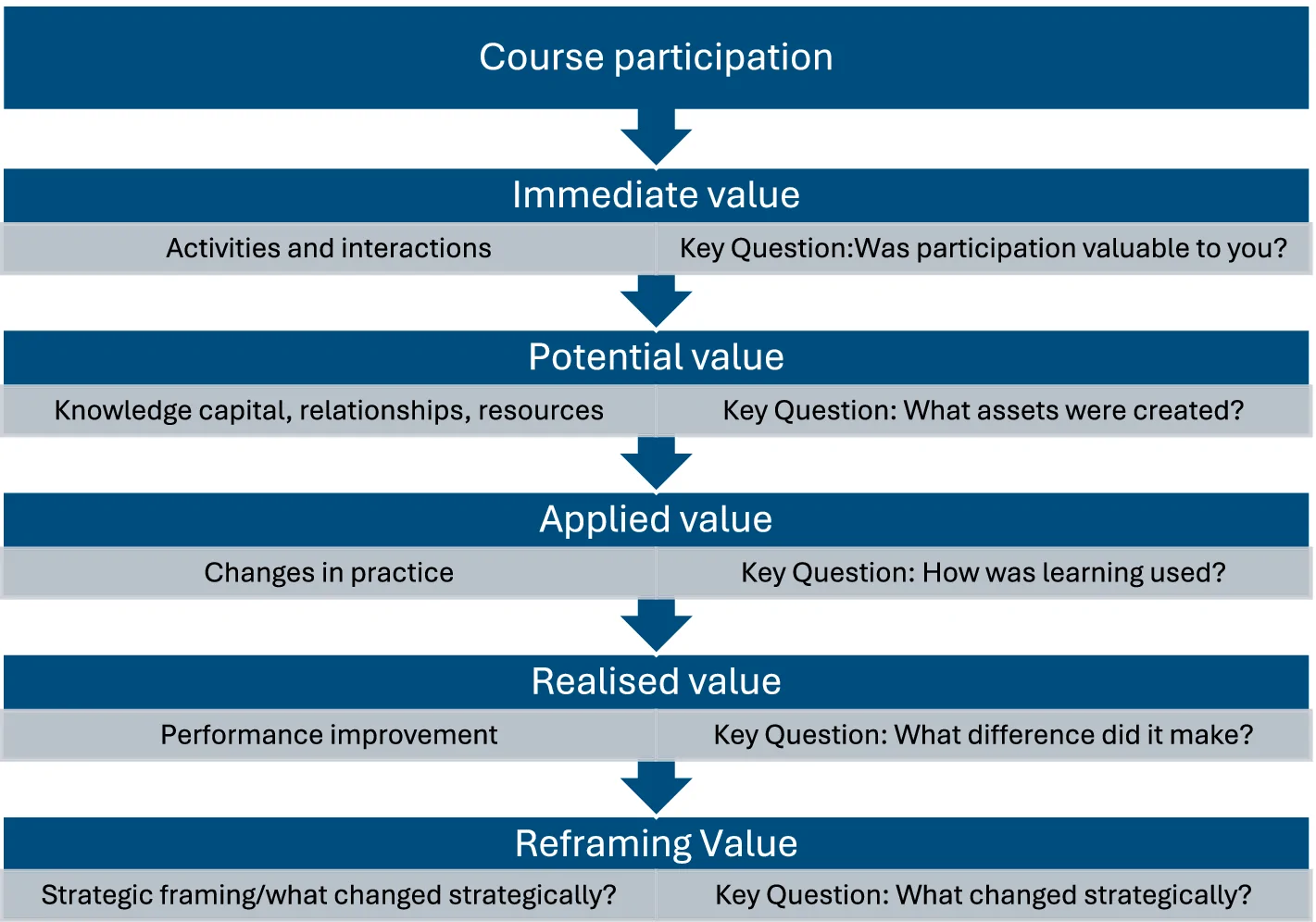
Adaptation of Wenger’s value creation framework and its application to course evaluation.

The study employed a convergent mixed-methods design (21), in which quantitative and qualitative data were collected during the same evaluation period, analysed separately, and integrated at the interpretation stage to generate complementary insights to evaluate the *Pandemics: Emergence, Spread and Response* short course. A participatory evaluation approach was used involving course participants as co-evaluators in shaping evaluation questions, contributing data and interpreting findings (22, 23). The evaluation was additionally informed by Wenger, Trayner and de Laat’s Value Creation Framework, which conceptualises learning as a process of value creation occurring across five interconnected cycles: immediate, potential, applied, realised and reframing value. The framework provided the conceptual structure for exploring how participants experienced the course, translated learning into practice, generated professional and organisational outcomes, and contributed to wider organisational or systems-level change. Accordingly, both the quantitative and qualitative data collection tools were designed to capture evidence across these different dimensions of value creation (19, 24). The framework also informed the analysis of participant narratives and integration of quantitative and qualitative findings (Figure 1).

### 2.2 Setting and participants

The evaluation population comprised all 50 individuals who completed the *Pandemics: Emergence, Spread and Response* course delivered between January and March 2022. The evaluation was conducted online between February and November 2023. All alumni were invited via personalised email to participate in the survey, focus group discussions, and workshop sessions; reminder emails were sent; participation was voluntary and individuals could participate in one or more components independently.

### 2.3 Participatory evaluation process

All 50 course participants were invited to join the evaluation team, of whom 13 volunteered. The team also included a learning designer, the UK-PHRST monitoring and evaluation (MEL) lead, and an educational research fellow. Over 8 months, the team convened for eight iterative workshops to refine evaluation questions, develop data collection tools, review preliminary results, and co-produce recommendations (25, 26) (Supplementary Table S2). Additional validation was conducted through a stakeholder validation workshop involving public health institutions from participants’ home countries.

### 2.4 Data collection

#### 2.4.1 Qualitative

Qualitative data were collected through two online focus group discussions (FGDs), each lasting approximately 60–90 min, and through Value Creation Stories (VCS). Eleven of the 50 course participants volunteered to join the FGDs. Two focus groups were conducted, comprising six and five participants, respectively. The 11 participants came from a range of organisation and different countries, including Nigeria (4), Uganda (3), Australia, Kenya, Zambia and Pakistan (1 each). In terms of job roles, participants included doctors, epidemiologists, a community pharmacist, an infectious disease specialist and a water sanitation officer.

The FGD guide explored participants’ perceptions of course usefulness, value, outcomes and impact on their personal development and/or professional practice The UK-PHRST MEL lead and learning designer conducted the FGDs. Discussions were recorded and transcribed verbatim during a Teams call. Transcripts were imported into NVivo (version 14) and analysed thematically following Braun and Clarke’s six-phase approach (27). Two evaluators independently coded an initial set of transcripts, resolving discrepancies by consensus. VCS were collected using a structured narrative approach derived from Wenger’s Value Creation Framework (19, 24). The approach enables participants to describe how learning experiences generate value over time and across different contexts. Participants were invited to reflect on: (1) specific course activities they found valuable (immediate value); (2) knowledge, skills, resources, or relationships gained through participation (potential value); (3) how these were applied in their professional practice (applied value); (4) the outcomes or improvements that resulted from these changes in practice (realised value); and (5) whether the learning led to broader shifts in understanding, priorities, strategy, or organisational approaches (reframing value). This structure facilitated exploration of learning trajectories extending beyond course completion and supported identification of individual, organisational, and system-level outcomes (Supplementary materials text S1).

#### 2.4.2 Quantitative

Quantitative data were collected using an online, self-administered questionnaire administered between 29 May and 9 June 2023 using SurveyMonkey. Survey settings were configured to limit participants to a single response. Participation was voluntary, and responses were collected anonymously. No identifying information was collected as part of the questionnaire, and survey responses were analysed in aggregate (*n* = 31; response rate 62%). The survey comprised 22 questions covering participant demographics, course experience, satisfaction, engagement levels, and perceived impact. Items were measured using structured Likert-type response categories as well as open-ended questions. Thirty-one participants completed the questionnaire. Missing responses were minimal and occurred for only a small number of survey items. Analyses were conducted using complete-case analysis for each variable; percentages were calculated using the number of respondents who answered each question.

The primary outcome variable was perceived course usefulness, defined as participants’ overall assessment of the extent to which the course supported their professional practice. This outcome was selected as an indicator of learning relevance and potential for application in complex professional contexts. It was measured by asking learners to rate the course as “useful,” “somewhat useful,” or “not useful.” Owing to the small sample size and sparse distribution across the latter two categories (2 and 3 responses, respectively), the outcome was dichotomised for regression analyses into “useful” versus “somewhat useful/not useful.” This approach enabled stable estimation while distinguishing participants reporting unequivocal course usefulness from those reporting more limited perceived benefit. Key explanatory variables included satisfaction with course content and materials, the learning platform, delivery mode, facilitator diversity, and engagement. Engagement was measured using participants’ self-reported level of engagement with the course, based on survey items assessing active participation in learning activities, interaction with facilitators and peers, and overall involvement during the course. We recognise that engagement may function both as an outcome of instructional design and as a determinant of perceived course usefulness. In our exploratory analyses, engagement was treated as an explanatory variable because we sought to examine whether participants reporting higher levels of engagement were also more likely to perceive the course as useful.

### 2.5 Data analysis

#### 2.5.1 Qualitative analysis

Qualitative data from FGDs and VCS narratives were analysed using reflexive thematic analysis following Braun and Clarke’s six-phase approach (27). Coding and theme development were conducted inductively to identify emerging patterns in the data using NVivo (version 14). Coding was undertaken independently by two researchers, followed by iterative discussion to achieve consensus. Ongoing input from the evaluation team was provided to enhance validity and contextual interpretation and accuracy. Once the themes had been developed, they were mapped deductively onto Wenger’s Value Creation Framework to examine how participants described different forms of value creation (19, 28, 29). This approach enabled the evaluation to identify not only participants’ experiences of the course but also the pathways through which learning contributed to changes in practice, professional performance, organisational development and broader public health responses. Rather than seeking theoretical saturation, the qualitative component aimed to capture diverse participant experiences across complementary qualitative methods. Convergence of findings across focus groups, VCS and survey responses strengthened confidence in the final themes.

#### 2.5.2 Quantitative analysis

Descriptive statistics summarised respondent characteristics and course experiences. Owing to the modest sample size, quantitative analyses were primarily descriptive. Associations between perceived course usefulness (useful vs. somewhat/not useful) and 20 explanatory variables were explored using Fisher’s exact tests since expected cell counts were small (30). Given the exploratory nature of the study, relatively small sample size, limited statistical power and the number of variables examined, no adjustment was made for multiple comparisons, and findings should be interpreted as hypothesis-generating rather than confirmatory. Quantitative findings were subsequently integrated with qualitative findings using Wenger’s Value Creation Framework to provide explanatory depth.

#### 2.5.3 Integration of findings

Findings were integrated at the interpretation stage through triangulation, comparing qualitative and quantitative results to identify convergence, complementarity and divergence across data sources (31). Integration focused on explaining how quantitative patterns were supported or elaborated by qualitative insights, particularly regarding mechanisms of learning transfer. Integration was also guided by Wenger’s Value Creation Framework to explore how evidence from different data sources reflected pathways of value creation from learning experiences through to changes in practice, realised outcomes and wider organisational and sector influence.

### 2.6 Ethical considerations

Ethical clearance was obtained from the Research Ethics Committee in the London School of Hygiene and Tropical Medicine, UK (LSHTM Ethics Ref: 284054). All participants received written information about the study objectives, procedures, potential risks, and benefits prior to participation. Informed consent was obtained electronically for the survey and verbally (with recording) for FGDs and workshops. Participation in the evaluation was voluntary, and respondents could withdraw at any stage without consequence. All data were anonymised to protect confidentiality. Survey responses were collected anonymously using SurveyMonkey, and no directly identifying information was collected. Focus group participants were known to one another because of the nature of group discussions; however, participants were reminded to maintain confidentiality and quotations were anonymised before analysis by removing identifying information such as names, institutions and countries where appropriate. Given the relatively small cohort, complete anonymity cannot be guaranteed. Data were stored on secure, password-protected LSHTM servers in accordance with institutional data protection policies and the UK General Data Protection Regulation (GDPR).

### 2.7 Reflexivity

The participatory evaluation approach raised potential risks of positive or confirmation bias, as a few members of the research team were involved in the design and delivery of the course, and some course participants also served as co-evaluators. We recognise that these dual roles may have influenced both the interpretation of findings and participants’ willingness to provide critical feedback, particularly given that participants were aware of the researchers’ involvement in course development and delivery. One member of the participatory evaluation team subsequently became a joint first author on this manuscript. This individual contributed to the co-design of the evaluation, interpretation of findings, and manuscript development while also bringing the perspective of a course participant. We recognise that this dual role may have introduced the potential for confirmation bias.

Several safeguards were implemented to mitigate these risks. First, participation in the evaluation team was voluntary and independent of participation in the survey, focus groups, and Value Creation Stories. All course alumni were invited to participate in the evaluation regardless of whether they joined the evaluation team, ensuring that the findings reflected a broad range of perspectives beyond the co-evaluators. Second, data collection explicitly encouraged honest reflection on both positive and negative experiences. Interview guides, workshop discussions, and Value Creation Story prompts invited participants to identify challenges, unintended consequences, barriers to applying learning, and recommendations for improvement rather than focusing solely on successes. Critical perspectives, including concerns regarding the limited representation of LMIC faculty and teaching materials, the timing of the course, online delivery, and assessment approaches, were discussed during workshops and retained within the final thematic analysis. Third, triangulation across multiple qualitative and quantitative data sources (survey responses, focus group discussions, Value Creation Stories, and documentary review) enabled comparison of findings across methods and participants, reducing reliance on any single perspective. Coding and interpretation were undertaken iteratively by multiple members of the research team, with themes refined through discussion until consensus was reached. Finally, participatory validation workshops provided opportunities for participants to review and challenge emerging interpretations, and anonymous written feedback was used to capture perspectives that participants may have been less comfortable expressing during group discussions. These potential sources of bias are inherent to participatory evaluation, which deliberately values participants’ experiential knowledge and involvement in interpreting findings. Rather than seeking complete researcher detachment, our approach prioritised transparency, reflexivity, triangulation, and the inclusion of diverse and critical perspectives to enhance the trustworthiness of the findings.

## 3 Results

### 3.1 Quantitative findings

#### 3.1.1 Participant characteristics

All 50 course participants were eligible for inclusion; 31 completed the online survey (response rate 62%). Although non-response bias cannot be excluded, the respondent sample included participants from multiple countries, organisations, and professional disciplines, suggesting that a broad range of perspectives was represented. Eleven participants contributed to the FGDs, and seven submitted VCS narratives. Participation across components was voluntary and not mutually exclusive.

Survey respondents were predominantly men (65%; *n* = 20) and the remainder women. Five respondents (16%) reported having a disability—three with visual impairments and two with motor or physical difficulties. Most participants were based in Africa (77%, *n* = 24) and represented a diverse range of professional settings, most commonly government ministries (36%; *n* = 11) or non-governmental organisations (29%; *n* = 9; Table 1).

**Table 1:** Baseline characteristics of survey respondents (*n* = 31).

Characteristic	n (%)
Sex	
Male	20 (65)
Female	11 (35)
Disability status	
Yes	5 (16)
No	26 (84)
Region	
Africa	24 (77)
Other regions including Australia (1), America (2), Asia (2) and Europe (2)	7 (23)
Primary organisation	
Government ministry	11 (36)
NGO	9 (29)
Other (UN agencies, pharmaceutical industry, medical research institute, intergovernmental organisations)	11 (35)

#### 3.1.2 Course experience and satisfaction

**Figure 2:**
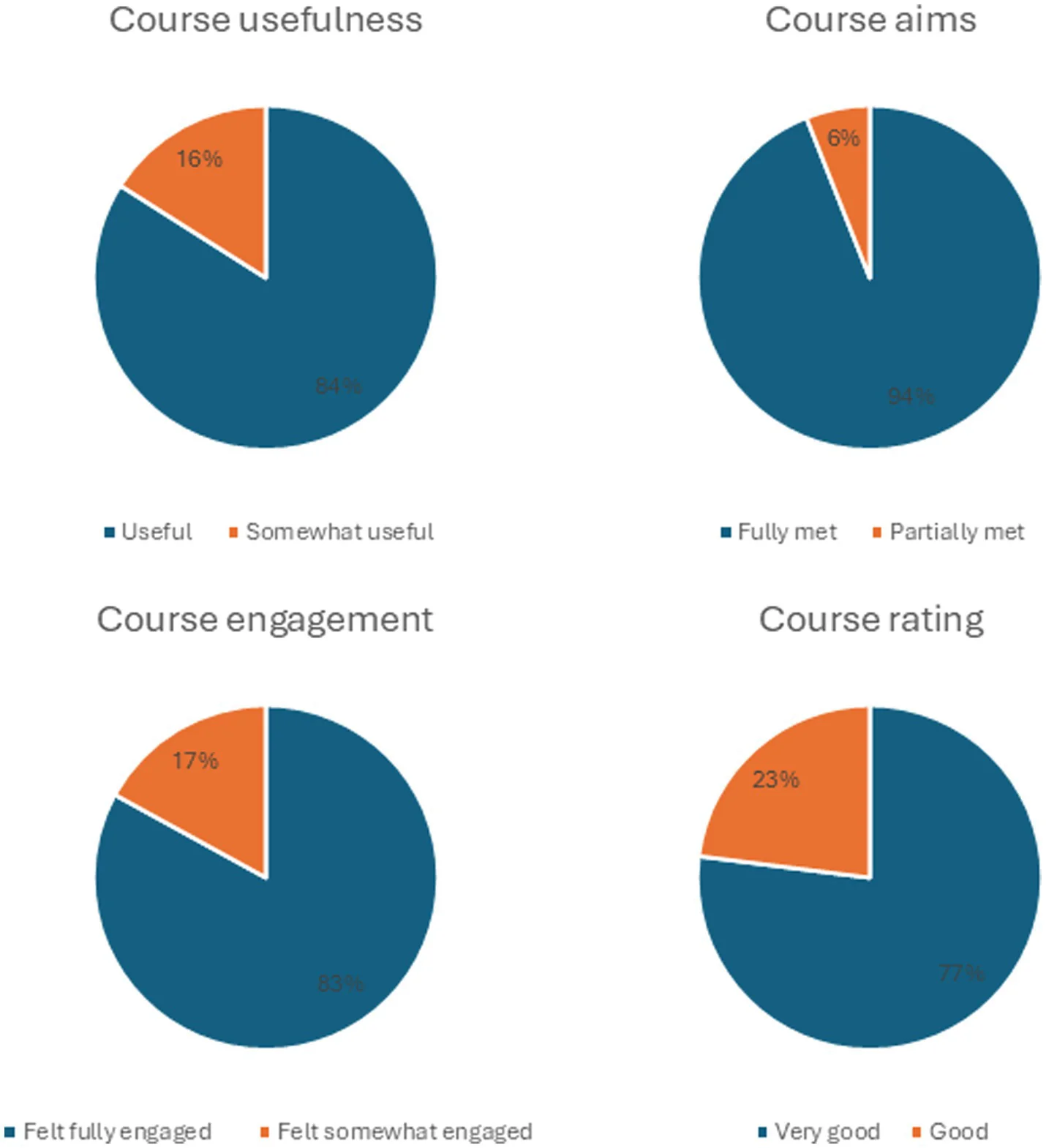
Course usefulness, aims, engagement and rating as perceived by course participants.

All survey respondents reported that the course was relevant to professionals working in disease outbreak contexts. Overall ratings of usefulness, clarity of aims, engagement and course quality were high (Figure 2). Overall, 26/31 (83.9%) participants rated the course as useful, 2/31 (6.5%) as somewhat useful, and 3/31 (9.7%) as not useful.

**Figure 3:**
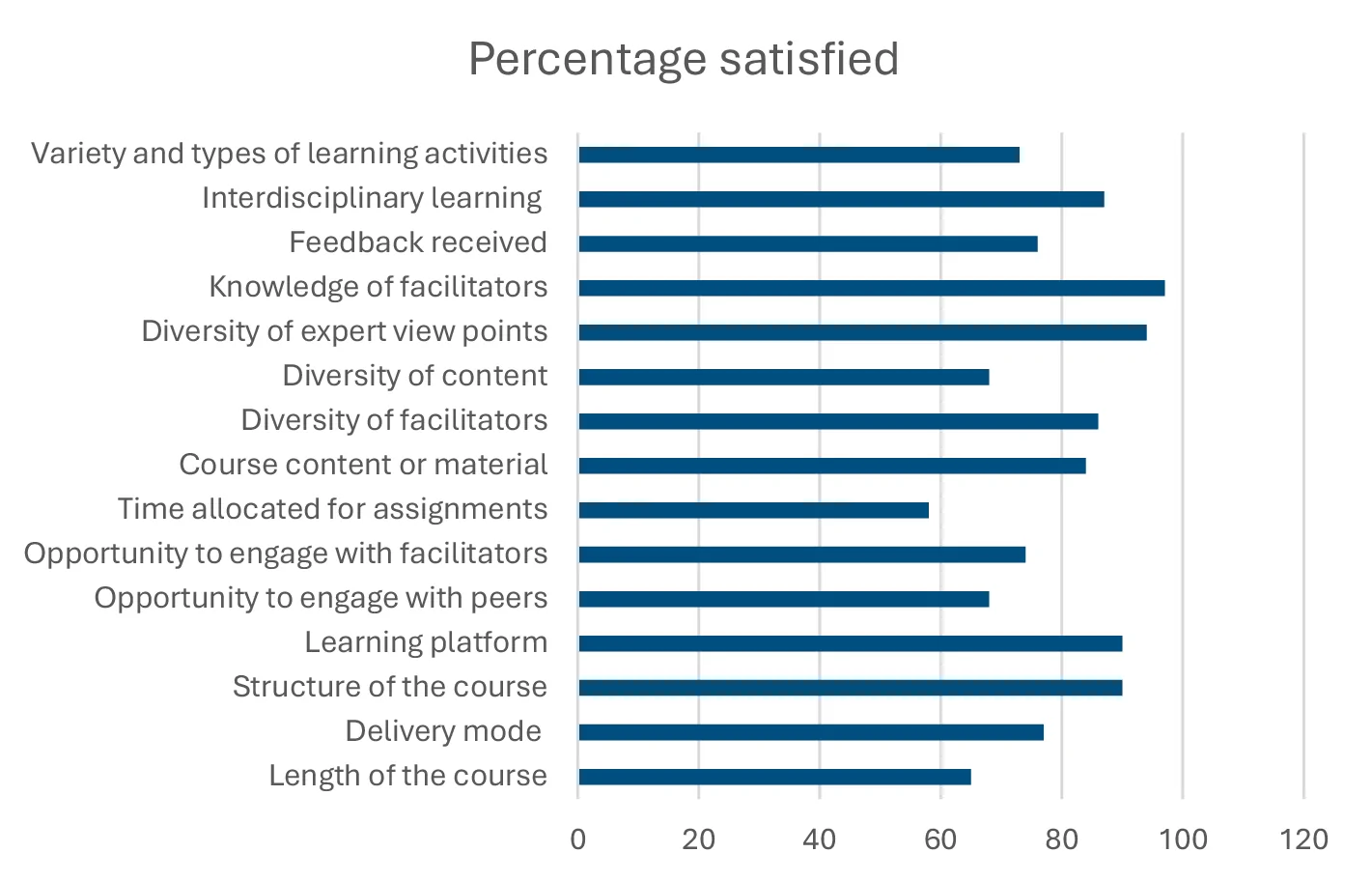
Learner satisfaction with the ‘pandemics: emergence, spread and response’ short course.

Satisfaction across course components was also generally high (Figure 3), particularly for facilitator expertise (97%), diversity of expert perspectives (94%), course structure and platform (90%). Lower satisfaction was reported for time allocated for assignments (58%), course length (65%) and opportunities for peer interaction (68%).

Participants with disabilities reported lower engagement and slightly lower perceived course quality compared with those without disabilities. Among participants with disabilities 40% (2/5) reported being “somewhat engaged,” compared with 12% (3/26) among those without, and 60% (3/5) rated overall course quality as “very good,” compared with 81% (21/26) of those without disabilities.

#### 3.1.3 Factors associated with perceived usefulness

Exploratory bivariate analyses suggested that participants reporting higher satisfaction with the mode of delivery, learning platform, course content, facilitator diversity and engagement were more likely to perceive the course as useful (Table 2). Given the modest sample size and exploratory analyses, these findings should be interpreted cautiously as indicative patterns rather than definitive associations.

**Table 2:** Exploratory associations between participant and course characteristics and perceived course usefulness (Fisher’s exact tests).

Predictor variable	Course had limited usefulness n (%)	Course was useful n (%)	Fisher’s exact *p*-value
Course characteristics			
Length of the course (10 weeks)			0.322
No	3 (27)	2 (10)	
Yes	8 (73)	18 (90)	
Mode of delivery (15 h asynchronous, 5 h synchronous)			**0.016**
No	3 (43)	2 (8)	
Yes	4 (57)	22 (92)	
Satisfaction with the structure of the course			0.289
No	1 (33)	4 (14)	
Yes	2 (67)	24 (86)	
Learning platform			**0.006**
No	2 (67)	3 (11)	
Yes	1 (33)	25 (89)	
Opportunity to engage with peers			0.599
No	1 (10)	4 (19)	
Yes	9 (90)	17 (81)	
Opportunity to engage with facilitators			0.157
No	0 (0)	5 (22)	
Yes	8 (100)	18 (78)	
Time allocated for assignments			0.352
No	3 (23)	2 (11)	
Yes	10 (77)	16 (89)	
Course content/material			**0.003**
No	3 (60)	2 (8)	
Yes	2 (40)	24 (92)	
Diversity of facilitators			**0.019**
No	2 (50)	2 (8)	
Yes	2 (50)	23 (92)	
Diversity of content			0.785
No	2 (20)	3 (14)	
Yes	8 (80)	18 (86)	
Diversity of expert views			0.187
No	1 (50)	4 (14)	
Yes	1 (50)	25 (86)	
Knowledge of facilitators			0.864
No	0 (0)	5 (17)	
Yes	1 (100)	25 (83)	
Quality of feedback			0.182
No	2 (29)	2 (9)	
Yes	5 (71)	20 (91)	
Interdisciplinary learning in international study groups			0.714
No	1 (25)	4 (15)	
Yes	3 (75)	23 (85)	
Course quality as a whole			0.457
No	1 (33)	4 (15)	
Yes	2 (67)	23 (85)	
Variety and types of learning activities			0.542
No	2 (25)	3 (14)	
Yes	6 (75)	19 (86)	
Full engagement with the course			**0.001**
No	4 (80)	1 (4)	
Yes	1 (20)	24 (96)	
Participant characteristics			
Sex			0.856
Female	2 (18)	3 (15)	
Male	9 (82)	17 (85)	
Disability status			0.255
No	3 (12)	2 (40)	
Yes	23 (88)	3 (60)	
Experienced disruption during study			0.219
No	2 (33)	3 (12)	
Yes	4 (67)	22 (88)	

Bold values indicate the statistically significant results of *p*-values.

### 3.2 Qualitative findings

Themes were developed inductively from the data and subsequently mapped onto Wenger’s Value Creation Framework to examine how different forms of value were generated through participation in the course. Thematic analysis identified five overarching themes and multiple related subthemes that described participants’ experiences of the course, its application to professional practice, and recommendations for future improvement (Table 3). The themes encompassed the most and least valuable aspects of the course, factors enabling the application of learning, impacts on work and career, application of learning in professional contexts, and suggested improvements to future course delivery. Although the thematic analysis generated five inductive themes relating to participants’ experiences of the course (Table 3), these findings were subsequently interpreted using Wenger’s Value Creation Framework to better understand how learning translated into professional practice over time. The following sections therefore present the qualitative findings according to Wenger’s five value cycles, illustrating the progression from participants’ immediate learning experiences to longer-term professional, organisational and systems-level impacts.

**Table 3:** Summary of themes and subthemes identified through thematic analysis of participants’ experiences and perceived impacts of the course.

Themes	Subthemes
Improvements to the course	More diversity
	Assessments
	Mode of delivery
	Timing
	Increased interaction
	Status of course
	Course fees & benefits
Least valuable aspects of the course	Insufficient representation of LMICs peers course material /teaching
	Assessments approach
	Timing
	Limited quality of online
Impact on work and career	Prestige of course
	Shaping careers or career direction
	Changed career paths
	Improved knowledge and skills
	Introduced policy changes
	Improved networks
	Greater questioning attitude
	Knowledge of better management of pandemics
Application to work contexts	Developing & influencing policy
	Improved structure/systems
	Better planning
	Disseminating learning more widely
	Application to teaching
Most valuable aspects of the course	Diversity of participants
	Diversity of experts
	Cross sectional
	Encouraging collaboration
	Shaping career
	Assessment
Enabling the application of learning	Knowledge & skills gained
	Practical examples & case studies
	Relatable, real life examples
	Simulation exercises & panel group discussions

#### 3.2.1 Immediate value: most valuable aspects of the course

Participants described three main sources of immediate value:

(1) Relevance of course content:

Course materials were described as practical and directly applicable to participants’ work.

*‘The real-life examples were relatable and can be directly applied in the context I work in.’*

(2) Facilitator expertise:

Participants valued the applied experience of teaching staff.

*‘The teachers were experts in their fields … this meant the lectures were also focused on real-*world *examples.’*

(3) Diversity of perspectives

While valued overall, participants noted a perceived bias towards western perspectives and expressed a desire for greater representation from LMICs-based experts.

*“I think there was a huge bias towards the west. So, I think in as much there are still lessons learned from there, you would want to learn more from peers who are in a low-income setting, whether Asia or Africa.’*

#### 3.2.2 Potential value: knowledge, skills and professional networks

Participants reported developing:

Professional credibility as institutional experts in pandemic preparedness and response:

*“I’m getting this sort of recognition in my department that you are like the specialist in this… it’s really given me that sort of credibility.”*

*“So, my colleagues know I have this [expertise]… and wherever there is need it’s me they call upon to train my colleagues.”*

Strengthened technical and analytical skills:

*“Learning on job and all that, and taking up this course, made me become so strong in terms of what I’m doing. And now you know, if they called me to respond on outbreak, I’m so sure that I have something to back me up.”*

*“Learning [on the course] about epidemic intelligence, translation and sharing of data was instrumental in the development of a cholera national policy.’*

*“After the course, whenever we want to embark on active case search, we begin to ask ourselves questions, in terms of capacity, and capabilities, and resources. How do we ensure that the available resources are allocated where they are supposed to be? Even, we’ll come back and say, let us do a review…let us do a sort of debriefing? What went well, what did not go well? This informs our subsequent planning.”*

Professional networks:

*"One of the key things was creating networks, and also just gaining experience from people from different backgrounds and environments".*

*“Immediately after the course, I think we are like that in a class across the globe. And then…will publish a paper that is looking at lessons learned from Ebola, COVID-19, we did that paper where 10 of us did that paper. And it cuts across the globe. And that, that begins to build synergy, that begins to build partnership collaboration, at a global level.”*

#### 3.2.3 Applied value: application of learning in professional practice

Participants reported applying course learning across three main domains:

Policy development:

*“The course contents was so relevant to me, because we had to improve border health. And my input was necessary. So for policy changes, for COVID by the Minister of Health, I gave him my input. And it was linked to the pandemic emergency response.”*

Systems and structural improvements:

Knowledge and skills gained strengthened institutional processes such as documentation and preparedness systems:

*“[We are] organising certain things better, like in the Public Health Emergency Operations Center. One of the things from the course is organisational capability. So, putting certain aspects in place…most of the documents, say, Standard Operating Procedures, the field handbook, implementations plans that were not [previously] in place… I think a lesson from the course is just having documentation in place.”*

*Because of the awareness [skills] I gained, I was motivated to carry out measures to mitigate this problem [microbial infection] at my workplace.”*

Knowledge sharing and capacity strengthening:

Participants shared their learning with colleagues through peer training, mentorship, and institutional briefings, extending the course’s reach and reinforcing professional networks. Course learning was therefore translated into practice across operational, organisational and policy contexts.

*“I have applied the knowledge gain(ed) to contribute as a co-author in a relevant publication, and applied the knowledge in the team I work especially when seeing my patients.”*

*“It’s me they call upon to train my colleagues.”*

#### 3.2.4 Realised value: professional and organisational outcomes

Realised value captures improvements resulting from applying learning in practice. Participants described a range of professional and organisational outcomes:

Career progression and leadership

*“I was promoted and tasked with mentoring the newly established Rapid Response Teams.”*

*“Embarking on the course has helped me to develop leadership skills.”*

Improved emergency response

*“The knowledge and skills I gained from the course were directly put into practice when my country was hit with an Ebola outbreak.”*

Institutional improvements

*“Learning about epidemic intelligence, translation and sharing of data was instrumental in the development of a cholera national policy.”*

#### 3.2.5 Reframing value: changes in perspectives and strategic thinking

The highest level of value creation occurred when learning fundamentally altered participants’ understanding of public health challenges and influenced strategic thinking. Several participants described shifts in how they conceptualised preparedness, collaboration and health systems.

One health thinking

*“Every day of my life now I think about collaboration*.”

Reconceptualising public health threats

*“Before taking the course, I had knowledge of antimicrobial resistance but never thought of it as a pandemic until it was highlighted as a silent pandemic during the course.”*

Strategic influence

*“I was invited to take part in the review of the country’s national infection prevention guidelines.”*

#### 3.2.6 Integration of findings

The qualitative findings provide explanatory depth for the exploratory quantitative findings by illustrating potential mechanisms through which participant engagement, content quality, facilitator diversity and interdisciplinary learning may have contributed to perceived course usefulness (Table 4). Integration of survey, focus group and Value Creation Story data suggests that perceived course usefulness extended beyond immediate learner satisfaction to influence professional practice, organisational processes, and broader public health systems. Consistent with Wenger’s Value Creation Framework, participants described a progression from immediate value derived from engaging content, expert facilitation, and interactive learning, to development of new knowledge, confidence and professional networks (potential value), the application of learning in workplace settings (applied value), improvements in professional practice and organisational preparedness (realised value), and changes in learners’ understanding of public health challenges, collaboration, preparedness, and systems thinking (reframing value). While the quantitative findings indicated high overall levels of perceived usefulness, the qualitative data also identified areas for improvement, including greater representation of LMIC perspectives, enhanced interaction, and refinements to assessment and course delivery. Together, these complementary findings suggest that the course generated value at individual, organisational and broader public health system levels, although these observations should be interpreted in light of the exploratory nature of the quantitative analyses.

**Table 4:** Joint display integrating quantitative and qualitative findings using Wenger’s value creation framework.

Quantitative finding	Qualitative explanation	Wenger’s value cycle
Higher participant engagement may be associated with greater perceived course usefulness	Interactive discussions, simulation exercises, and practical case studies enhanced learner engagement and facilitated learning	Immediate value
Greater facilitator diversity may be associated with higher perceived course usefulness	Participants valued facilitators’ real-world expertise, interdisciplinary perspectives, and practical experience	Immediate value
Greater perceived practical relevance may support course usefulness and application of learning	Real-life examples and case studies enabled participants to apply learning within their professional contexts	Applied value
Networking opportunities may contribute to perceived course usefulness	Participants developed new professional networks, collaborations, and peer learning opportunities	Potential value
Higher perceived course usefulness may facilitate workplace implementation	Participants reported influencing policy, strengthening preparedness systems, delivering training, and improving outbreak response	Realised value
Participants reporting greater course usefulness also described longer-term professional impacts	Participants described changes in leadership, systems thinking, and approaches to collaboration and pandemic preparedness	Reframing value

## 4 Discussion

This participatory mixed-methods evaluation suggests that an interdisciplinary online course on pandemic preparedness generated value extending beyond immediate learning experiences to influence professional practice, organisational processes, and broader public health preparedness. Guided by Wenger’s Value Creation Framework, the evaluation demonstrated a progression from immediate learning experiences through the development and application of knowledge to longer-term professional, organisational, and systems-level outcomes. By integrating survey findings, focus group discussions and Value Creation Stories, the study provides a richer understanding of how educational interventions may contribute to public health workforce development than evaluations relying solely on learner satisfaction or knowledge gain.

Previous evaluations of pandemic preparedness and emergency response training have predominantly focused on short-term outcomes, including learner satisfaction, perceived knowledge gain, confidence, competency development, or immediate post-course performance. For example, evaluations of the WHO OpenWHO COVID-19 vaccination training and the Infection Prevention and Control MOOC primarily assessed learner satisfaction, perceived knowledge gains, course completion and confidence following training, while earlier preparedness programmes similarly relied on pre- and post-test measures of knowledge or behavioural intentions (32–34). A recent scoping review likewise concluded that although most training programmes evaluated participant knowledge, skills and satisfaction, relatively few examined whether learning translated into sustained professional practice, organisational change or broader preparedness capacity (35). In contrast, the present study adopted Wenger’s Value Creation Framework to explore how learning was mobilised over time within participants’ professional contexts, extending evaluation beyond immediate educational outcomes to include workplace application, organisational development and systems-level influence.

Using Wenger’s framework as an organising structure, the findings suggest that value creation occurred across all five interconnected cycles. Immediate value was reflected in participants’ positive experiences of relevant content, expert facilitators and opportunities for interdisciplinary interaction. Potential value emerged through the development of technical knowledge, professional confidence and expanded networks, which participants perceived as enhancing their credibility and preparedness for future outbreak response. Applied value was evident where participants described adapting and using course learning within their own professional contexts, including policy development, preparedness planning, teaching and emergency response activities. Participants further described examples of realised value, including leadership opportunities, strengthened organisational processes and contributions to preparedness systems. Finally, reframing value was reflected in broader changes in participants’ understanding of collaboration, One Health, systems thinking and pandemic preparedness, suggesting that the course influenced not only what participants knew, but also how they conceptualised complex public health challenges. These findings are consistent with previous research suggesting that interdisciplinary and problem-oriented educational approaches strengthen collaborative capability, adaptive expertise and systems thinking required for complex public health emergencies (1, 2, 36). These findings additionally align with studies showing that interactive pedagogies and real-world relevance are critical for sustaining learning outcomes in emergency preparedness training (37–39). The prominence of One Health and cross-sectoral thinking among participants also reflects increasing recognition that effective pandemic preparedness depends on collaboration across disciplines, sectors and geographical settings (36, 40).

The integration of quantitative and qualitative findings provides complementary evidence regarding the mechanisms through which value may have been created. The exploratory quantitative analyses suggested that participant engagement, perceived quality of course content, facilitator diversity and interactive learning were associated with greater perceived course usefulness. The qualitative findings provided explanatory depth by illustrating how these features supported learning through practical case studies, simulation exercises, interdisciplinary discussion and exposure to diverse professional perspectives. Participants consistently described these experiences as facilitating the application of learning within their workplaces and encouraging collaboration beyond the course itself. Given the modest sample size and exploratory nature of the quantitative analyses, these findings should be interpreted cautiously as descriptive patterns that complement the qualitative findings rather than as evidence of causal relationships. These findings are consistent with evidence from online and blended professional education demonstrating that learner engagement, high-quality instructional design, interactive pedagogies and facilitator expertise are central determinants of meaningful learning experiences (41–44). They also reinforce recent evidence that simulation exercises, authentic case studies and collaborative learning support the transfer of knowledge into professional practice (45, 46).

The study also highlights the importance of evaluating educational interventions beyond immediate educational outcomes. Traditional evaluation frameworks often focus on learners’ reactions or short-term knowledge acquisition, whereas Wenger’s Value Creation Framework enabled exploration of how learning evolved over time into workplace application, organisational change and broader systems thinking. The framework proved particularly valuable for understanding the dynamic processes through which professional education contributes to public health workforce development and preparedness capacity. The conceptual pathway proposed in Figure 1 illustrates how key course design features, including interdisciplinary content, interactive pedagogy, expert facilitation and diverse participant engagement, may support progression across successive cycles of value creation from learning to organisational and systems-level impact.

Several lessons for future course development also emerged. Participants emphasised the importance of increasing representation of experts and case studies from low- and middle-income countries to enhance contextual relevance and ensure that diverse experiences of epidemic preparedness are reflected within the curriculum. A small number of participants who self-identified as having a disability also highlighted accessibility challenges. Given the limited number of respondents, these observations should be interpreted cautiously and viewed as lessons for improving future programmes rather than definitive findings. Together, they reinforce the importance of designing inclusive learning environments that accommodate diverse learners and reflect a broader range of global perspectives (20, 47). These observations align with growing recognition that global health education should move beyond predominantly high-income country perspectives towards more equitable and contextually relevant curricula that better reflect the realities of pandemic preparedness in low- and middle-income settings (48, 49). Similarly, accessibility has become an increasingly recognised consideration in online professional education, although empirical evidence remains limited (20).

The participatory evaluation approach represents both a strength and a limitation of this study. Involving alumni as co-evaluators strengthened evaluation design, delivery and interpretation of findings by incorporating participants’ experiential knowledge and supporting collective reflection on how learning generated value over time. The inclusion of Value Creation Stories further enabled the evaluation to capture outcomes that are often overlooked by conventional educational evaluations. These findings are consistent with previous work demonstrating that participatory evaluation enhances the relevance, utilisation and organisational ownership of evaluation findings (50). Beyond improving evaluation quality, involving participants as co-evaluators may itself represent a form of capacity strengthening by developing skills in critical reflection, evaluation and evidence-informed decision-making (51). However, participatory evaluation is resource-intensive and introduces potential risks of selection, confirmation and social desirability bias. Although these risks were mitigated through reflexive practice, triangulation across multiple data sources, independent coding, participant validation and the inclusion of critical perspectives, they cannot be eliminated entirely and should be considered when interpreting the findings.

The findings have several practical implications for the design and evaluation of future pandemic preparedness programmes. Course designers should prioritise interdisciplinary curricula, interactive pedagogical approaches, simulation exercises and opportunities for peer learning, while ensuring greater representation of faculty, case studies and lived experiences from low- and middle-income settings. Institutions delivering professional education should support the continued application of learning through communities of practice, mentoring and opportunities for workplace implementation beyond course completion. Funders should consider supporting longer-term evaluations that examine professional, organisational and systems-level outcomes rather than relying solely on immediate measures of learner satisfaction or knowledge gain. Finally, future evaluations should incorporate accessibility considerations from programme design through to evaluation and actively involve diverse stakeholders in assessing how educational interventions create value across different contexts.

Several limitations should be considered when interpreting these findings. The primary outcome was dichotomised because of the small number of participants selecting the “somewhat useful” and “not useful” categories, reducing the granularity of the original ordinal measure. The quantitative analyses were exploratory and descriptive in nature, and the observed patterns should be interpreted cautiously given the modest sample size and integrated with the qualitative findings rather than viewed as evidence of causal relationships. Outcomes were primarily based on self-reported experiences collected approximately 1 year after course completion, introducing the possibility of recall and social desirability bias. Participation in the evaluation was voluntary, and non-response bias cannot be excluded. Given the relatively small cohort, complete anonymity could not be guaranteed. Furthermore, although participants described substantial professional and organisational impacts, attribution remains challenging given the complex organisational and policy environments in which public health professionals operate. Only seven alumni submitted Value Creation Stories, and these narratives should therefore be regarded as illustrative rather than representative of the wider participant group. Nevertheless, the convergence of evidence across surveys, focus groups and Value Creation Stories strengthens confidence in the overall interpretation of the findings and demonstrates the utility of Wenger’s Value Creation Framework for evaluating complex educational interventions.

This study suggests that the value of pandemic preparedness training extends beyond immediate educational outcomes to encompass changes in professional practice, organisational development and broader preparedness systems. By combining participatory evaluation methods with Wenger’s Value Creation Framework, the evaluation captured pathways of learning transfer that are frequently overlooked by conventional educational evaluations focused primarily on learner satisfaction or knowledge acquisition. The findings suggest that future pandemic preparedness programmes should prioritise interdisciplinary learning, interactive pedagogies, practical case-based teaching, opportunities for workplace application, and sustained peer networks that support continued learning beyond course completion. Strengthening accessibility and increasing representation of expertise and experiences from low- and middle-income countries are likely to enhance both the relevance and inclusiveness of future programmes. For institutions and funders investing in public health workforce development, these findings emphasise the importance of evaluating educational interventions using measures that extend beyond immediate learner outcomes to include changes in professional practice, organisational capacity and preparedness systems. Wenger’s Value Creation Framework provides a practical and transferable conceptual approach for understanding these longer-term pathways of impact and for informing the design, delivery and evaluation of future capacity-strengthening initiatives.

## Data Availability

The raw data supporting the conclusions of this article will be made available by the authors, without undue reservation.

## References

[1] Holmes EA O'Connor RC Perry VH Tracey I Wessely S Arseneault L. Multidisciplinary research priorities for the COVID-19 pandemic: a call for action for mental health science. Lancet Psychiatry. (2020) 7:547–60. doi: 10.1016/S2215-0366(20)30168-1, 32304649 PMC7159850

[2] Ryan BJ Swienton R Harris C James JJ. Environmental health workforce - essential for interdisciplinary solutions to the COVID-19 pandemic. Disaster Med Public Health Prep. (2021) 15:e1–3. doi: 10.1017/dmp.2020.242, 32660683 PMC7403748

[3] LSHTM Pandemics: Emergence, spread and Response. UAE, Jordan: YouApply (2021)

[4] Raftery P Hossain M Palmer J. An innovative and integrated model for global outbreak response and research - a case study of the UK public health rapid support team (UK-PHRST). BMC Public Health. (2021) 21:1378. doi: 10.1186/s12889-021-11433-0, 34247621 PMC8273030

[5] Raftery P Hossain M Palmer J. A conceptual framework for analysing partnership and synergy in a global health alliance: case of the UK public health rapid support team. Health Policy Plan. (2022) 37:322–36. doi: 10.1093/heapol/czab150, 34919688 PMC9383178

[6] Kay D Kibble J. Learning theories 101: application to everyday teaching and scholarship. Adv Physiol Educ. (2016) 40:17–25. doi: 10.1152/advan.00132.2015, 26847253

[7] Chandramohan B Fallows S. Interdisciplinary Learning and Teaching in Higher Education. Abingdon, UK: Routledge (2009).

[8] Jones C. Interdisciplinary approach - advantages, disadvantages, and the future benefits of interdisciplinary studies. ESSAI. (2010) 7:28. Available online at: http://dc.cod.edu/essai/vol7/iss1/26

[9] Klaassen RG. Interdisciplinary education: a case study. Eur J Eng Educ. (2018) 43:842–59. doi: 10.1080/03043797.2018.1442417

[10] Lindvig K Ulriksen L. Different, difficult, and local: a review of interdisciplinary teaching activities. Rev High Educ. (2019) 43:697–725. doi: 10.1353/rhe.2019.0115

[11] Findyartini A Greviana N Hanum C Husin JM Sudarsono NC Krisnamurti DGB. Supporting newly graduated medical doctors in managing COVID-19: an evaluation of a massive open online course in a limited-resource setting. PLoS One. (2021) 16:e0257039. doi: 10.1371/journal.pone.0257039, 34506524 PMC8432880

[12] Ashcroft J Byrne MHV Brennan PA Davies RJ. Preparing medical students for a pandemic: a systematic review of student disaster training programmes. Postgrad Med J. (2020) 97:368–79. doi: 10.1136/postgradmedj-2020-137906, 32518075 PMC7316122

[13] Meng W Yu L Liu C Pan N Pang X Zhu Y. A systematic review of the effectiveness of online learning in higher education during the COVID-19 pandemic period. Front Educ. (2024) 8:1334153. doi: 10.3389/feduc.2023.1334153

[14] Xu T Xue L. Satisfaction with online education among students, faculty, and parents before and after the COVID-19 outbreak: evidence from a meta-analysis. Front Psychol. (2023) 14:1128034. doi: 10.3389/fpsyg.2023.1128034 36860782 PMC9968937

[15] Nayahangan LJ Konge L Russell L Andersen S. Training and education of healthcare workers during viral epidemics: a systematic review. BMJ Open. (2021) 11:e044111. doi: 10.1136/bmjopen-2020-044111, 34049907 PMC8166630

[16] Boutros P Kassem N Nieder J Jaramillo C von Petersdorff J Walsh FJ. Education and training adaptations for health workers during the COVID-19 pandemic: a scoping review of lessons learned and innovations. Healthcare (Basel). (2023) 11:2902. doi: 10.3390/healthcare11212902, 37958046 PMC10649637

[17] Akinluyi EA Greenough A Ison K Clarkson PJ. Applying a participatory systems and value approach in a transdisciplinary exercise: on assessing the impact of training and education initiatives. Health Syst (Basingstoke). (2023) 12:446–60. doi: 10.1080/20476965.2023.2230632, 38235305 PMC10791087

[18] Cousins JB Alborhamy Y. Theory-practice connections in collaborative approaches to evaluation: a systematic review of practice. Am J Eval. (2025) 46:491–517. doi: 10.1177/10982140251355140

[19] Wenger E Trayner B De Laat M. Promoting and Assessing Value Creation in Communities and Networks: a Conceptual Framework. The Netherlands: Open University of the Netherlands (2011).

[20] Wild J Nzegwu F. Digital technology in capacity development: enabling learning and supporting change. African Minds. (2023). doi: 10.47622/9781928502708

[21] Palinkas LA Mendon SJ Hamilton AB. Innovations in mixed methods evaluations. Annu Rev Public Health. (2019) 40:423–42. doi: 10.1146/annurev-publhealth-040218-044215, 30633710 PMC6501787

[22] Chouinard JA Cousins JB. The journey from rhetoric to reality: participatory evaluation in a development context. Educ Assess Eval Account. (2015) 27:5–39. doi: 10.1007/s11092-013-9184-8

[23] Cousins JB Chouinard JA. Participatory Evaluation up close: An Integration of Research-based Knowledge. North Carolina, USA: IAP (2012).

[24] Menchaca M. Cowan J. “Value Creation Stories in a Community of Practice: Assessing Value in an Online masters program” (2014) *Proceedings of world conference on e-learning* New Orleans, LA, USA Association for the Advancement of Computing in Education (AACE) 1340–1349

[25] O’Cathain A. Workshops as a qualitative research method in health research. BMJ Open. (2025) 15:e106459. doi: 10.1136/bmjopen-2025-106459, 41005775 PMC12481299

[26] Ørngreen R Levinsen K. Workshops as a research methodology. Electron J E-Learn. (2017) 15:70–81. Available online at: www.ejel.org.

[27] Braun V Clarke V. Using thematic analysis in psychology. Qual Res Psychol. (2006) 3:77–101. doi: 10.1191/1478088706qp063oa

[28] Wenger-Trayner B Wenger-Trayner E Cameron J Eryigit-Madzwamuse S Hart A. Boundaries and boundary objects: an evaluation framework for mixed methods research. J Mixed Methods Res. (2019) 13:321–38. doi: 10.1177/1558689817732225

[29] Wenger-Trayner E Wenger-Trayner B. Learning to Make a Difference: Value Creation in Social Learning Spaces. Cambridge: Cambridge University Press (2020).

[30] Kim HY. Statistical notes for clinical researchers: chi-squared test and fisher's exact test. Restor Dent Endod. (2017) 42:152–5. doi: 10.5395/rde.2017.42.2.152, 28503482 PMC5426219

[31] Moseholm E Fetters MD. Conceptual models to guide integration during analysis in convergent mixed methods studies. Methodol Innov. (2017) 10:2059799117703118. doi: 10.1177/2059799117703118

[32] Espinoza-Castro B Encina V Garrido MA Vinueza FI Piedra JP Garzon-Villalba X. Online learning for crisis response: evaluating reach and perceived knowledge gains from the MOOC “infection, prevention, and control of acute respiratory infections for healthcare Workers in low- and Middle-Income Countries (IPC MOOC)”. BMC Med Educ. (2025) 25:1150. doi: 10.1186/s12909-025-07661-2, 40775697 PMC12330176

[33] Gershon RR Vandelinde N Magda LA Pearson JM Werner A Prezant D. Evaluation of a pandemic preparedness training intervention of emergency medical services personnel. Prehosp Disaster Med. (2009) 24:508–11. doi: 10.1017/S1049023X00007421, 20301068

[34] Goldin S Kong SYJ Tokar A Utunen H Ndiaye N Bahl J. Learning from a massive open online COVID-19 vaccination training experience: survey study. JMIR Public Health Surveill. (2021) 7:e33455. doi: 10.2196/33455, 34794116 PMC8647976

[35] Burton K Best J DeCoster J Ritchwood TD. Pandemic ready or playing catch-up? A scoping review of public health training programs for pandemic preparedness and response efforts. BMC Public Health. (2026) 26:1687. doi: 10.1186/s12889-026-27090-0, 41987085 PMC13200470

[36] Interdisciplinary Research in Epidemic Preparedness and Response: Workshop Report. London, UK: The Academy of Medical Sciences (2019).

[37] Patrick SM Nicholas N Maritz M Wolvaardt JE. Enhancing public health education through smart learning environments: integrating technology and pedagogy. Med Sci Educ. (2025) 35:2249–56. doi: 10.1007/s40670-025-02408-6, 41112887 PMC12532516

[38] Ramírez-Mera UN Ramírez Díaz JA. From rapid response to future preparedness: a systematic review of tailored practices in emergency pedagogy during the COVID-19 era. Int Rev Educ. (2025) 72:277–300. doi: 10.1007/s11159-025-10163-4

[39] Utunen H Balaciano G Arabi E Tokar A Bhatiasevi A Noyes J. Learning interventions and training methods in health emergencies: a scoping review. PLoS One. (2024) 19:e0290208. doi: 10.1371/journal.pone.0290208, 39012917 PMC11251632

[40] One Health High-Level Expert Panel (OHHLEP) Adisasmito WB Almuhairi S Behravesh CB Bilivogui P Bukachi SA . One health: a new definition for a sustainable and healthy future. PLoS Pathog. (2022) 18:e1010537. doi: 10.1371/journal.ppat.1010537, 35737670 PMC9223325

[41] Alselaiti N. Enhancing student engagement and learning outcomes through education technologies in medical education. World J Adv Res Rev. (2023) 19:1356–67. doi: 10.30574/wjarr.2023.19.3.1922

[42] Armellini A Teixeira Antunes V Howe R. Student perspectives on learning experiences in a higher education active blended learning context. TechTrends. (2021) 65:433–43. doi: 10.1007/s11528-021-00593-w

[43] De Gagne JC Koppel PD Park HK Cadavero A Cho E Rushton S . Nursing students' perceptions about effective pedagogy: netnographic analysis. JMIR Med Educ. (2021) 7:e27736. doi: 10.2196/27736, 34156337 PMC8277346

[44] Verbree A-R Isik U Janssen J Dilaver G. Inclusion and diversity within medical education: a focus group study of students’ experiences. BMC Med Educ. (2023) 23:61. doi: 10.1186/s12909-023-04036-3, 36698110 PMC9875758

[45] Shah D Behravan N Al-Jabouri N Sibbald M. Incorporating equity, diversity and inclusion (EDI) into the education and assessment of professionalism for healthcare professionals and trainees: a scoping review. BMC Med Educ. (2024) 24:991. doi: 10.1186/s12909-024-05981-3, 39261856 PMC11391843

[46] Zhao T Deng W Yang Y Jin Y Xu F. Enhancing student satisfaction through open collaborative practical teaching reforms in public health education: a comparative study. Front Public Health. (2025) 13:1546962. doi: 10.3389/fpubh.2025.1546962, 40109428 PMC11919846

[47] CAST. Universal design for Learning Guidelines 3.0. Wakefield (MA): CAST (2024).

[48] Eichbaum QG Adams LV Evert J Ho MJ Semali IA van Schalkwyk SC. Decolonizing global health education: rethinking institutional partnerships and approaches. Acad Med. (2021) 96:329–35. doi: 10.1097/ACM.0000000000003473, 32349015

[49] Kumar R Khosla R McCoy D. Decolonising global health research: shifting power for transformative change. PLOS Glob Public Health. (2024) 4:e0003141. doi: 10.1371/journal.pgph.0003141, 38656955 PMC11042701

[50] Smits PA Champagne F. An assessment of the theoretical underpinnings of practical participatory evaluation. Am J Eval. (2008) 29:427–42. doi: 10.1177/1098214008325023

[51] Mayer K. "Reflexivity in co-evaluation: from challenges to principles of participatory research evaluation". In: Davies S R Schikowitz A Mora-Gámez F Goldberg E Dessewffy E Pham B-C , editors. Revisiting Reflexivity: Liveable Worlds in Research and Beyond (1st, First Edition). Bristol: Bristol University Press (2025). p. 273–81. doi: 10.2307/jj.18323787.26

