# Supplementary material for: Strengthening capacity for pandemic preparedness among health care professionals through interdisciplinary online learning: a participatory mixed-methods evaluation
Source: Front Public Health. 2026 Aug 26;14:1879989. doi: 10.3389/fpubh.2026.1879989 (PMC13553812; doi:10.3389/fpubh.2026.1879989)
Supplement: Supplementary file 1 [file Data_Sheet_1.PDF]

Supplementary Table S1: Overview of course structure

**Pandemics: Emergence, Spread and Response 10-week course overview**	
Part 1 – Introduction<br>*Introduction to emerging infectious diseases & history of epidemic response*	Week 1 – Definitions and drivers of emerging infectious diseases.
	Week 2 – Examining the history of epidemic containment to inform future pandemic response.
Part 2 – Prevent<br>*How can we prepare for & prevent pandemics?*	Week 3 – Past and future strategies to reduce pandemic risks from a prevention framework.
	Week 4 – Roles of global institutions and governance in pandemic preparedness.
Part 3 – Detect<br>*How are epidemics & pandemics detected?*	Week 5 – Detection of emerging infections & epidemics.
	Week 6 – Surveillance of emerging infections & declaring pandemics.
Part 4 – Respond<br>*How to coordinate a national pandemic response?*	Week 7 – Developing a multisectoral, One Health, national response for emerging zoonoses.
	Week 8 – Anticipating & addressing challenges to pandemic response.
Part 5 – The future<br>*How to better prepare for future pandemics?*	Week 9 – Engaging & supporting local responders to build resilient pandemic response: Sierra Leone.
	Week 10 – Preparing for future health crises: collaboration, coordination, innovation & equity.
