# Supplementary material for: Strengthening capacity for pandemic preparedness among health care professionals through interdisciplinary online learning: a participatory mixed-methods evaluation
Source: Front Public Health. 2026 Aug 26;14:1879989. doi: 10.3389/fpubh.2026.1879989 (PMC13553812; doi:10.3389/fpubh.2026.1879989)
Supplement: Supplementary file 2 [file Data_Sheet_2.PDF]

Supplementary Table S2: Details of the eight workshops held as part of the participatory evaluation.

Workshop No	Purpose	Participants
1	• Identified key thematic areas that the evaluation would address<br>• Conducted a stakeholder mapping	Evaluation Team
2	• Reviewed the draft sets of evaluation questions and agreed on modifications<br>• Identified a survey, focus group discussions (FGDs), and value creation stories as the most appropriate data collection tools	Evaluation Team
3	• Reviewed and refined the data collection tools using feedback from the pilot<br>• Agreed a data collection plan.	Evaluation Team
4 & 5	• Initial findings presented to the team<br>• Further areas of analysis explored	Evaluation Team
6	• Developed a draft set of recommendations for future courses based on the evaluation findings.	Evaluation Team
7	• Data validation session where evaluation findings and draft recommendations were presented and discussed.	• Evaluation Team<br>• Stakeholders from public health institutions of participants.
8	• Final recommendations agreed based on feedback from validation workshop.	Evaluation Team
