# Supplementary material for: Strengthening capacity for pandemic preparedness among health care professionals through interdisciplinary online learning: a participatory mixed-methods evaluation
Source: Front Public Health. 2026 Aug 26;14:1879989. doi: 10.3389/fpubh.2026.1879989 (PMC13553812; doi:10.3389/fpubh.2026.1879989)
Supplement: Supplementary file 3 [file Data_Sheet_3.PDF]

Supplementary material Text C1: Value Creation Story (VCS) in the Pandemics' Preparedness and Response course

Adapted Value Creations Story (VCS) (Wenger et al 2011) was used as a basis for developing a narrative account of the value generated by the Pandemics' Preparedness and Response course activities for the course participants. VCS is a conceptual framework for assessing value creation in learning communities.

Value creation is a perspective on social learning that reflects the aspiration to make a difference. Social learning creates value to the extent that it is recognized as improving the ability to make that difference (Wenger-Trayner, https://www.wenger-trayner.com/value-creation/)

The course facilitated social learning in several ways – by connecting learners to a global network of experts and practitioners in the field, encouraging collaboration between the participants, facilitating a debate on issues covered in the course, and supporting networking activities. The Value Creation Story helps to assess the extent to which the participation in the Pandemics' Preparedness and Response course has had an impact on i) a participant as an individual, ii) their own practice, iii) their organisation, and/or iv) their broader context.

A value-creation story follows a specific format (ibid 2011). The story is woven through each of the stages of value creation: immediate, potential, applied and realised value. The storyteller starts from describing a course activity that the learner benefited from. They reflect on the change to them as a person and a professional that occurred because of this activity (immediate value). The story then highlights a new idea, skill, relationship, resource that this activity generated for the storyteller (potential value). Next, it continues to describe how this idea, skill, relationship, or resource was used in practice and to what effect – what has it enabled them to do that would not have happened otherwise (applied value). Finally, the effect is linked to an outcome such as a performance indicator for the storyteller or the organisation or the broader context (realised value).

Data collection

An email was sent out to all participants of the course with a request to either complete an evaluation survey or VCS or participate in a focus group. Seven participants (five men and two women) completed the Value Creation Story templates. Timing of completing the VCS template is important especially when it comes to capturing the applied and realised value. A fair amount of time must elapse between the learning intervention and the opportunity to both apply the learning in the context of one's work and measure its success. The VCSs were collected 14 months after the completion of the course, so enough time has elapsed to trace applied and realised value for the course participants.

Limitations

The participants in the focus groups and value creation stories were self-selected, so already highly motivated. Six of the respondents were also part of the participatory evaluation team.

Value Creation Story Template

Thank you for volunteering to fill in this form. This Value Creation Story will help us to assess the extent to which your participation in the Pandemics' Preparedness and Response course has had an impact on you as a professional, your practice and your work in the pandemic context. You might only be able to fill in two of the steps in the table below but hopefully you will be able to fill in at least three or four. For step 5, you actually need to be able to measure the impact of your changed practice on a pandemic related situation in your work context. This might not have happened yet, but perhaps it has. Or it might happen soon, in which case you can always come back to us and fill in the final stage.

Conversely, if you feel you haven't benefited from any activities on this course, please use step one to one to tell us why.

Please read carefully through the instructions in each step, then provide a description of 100-200 words. If there are two or more activities that you have benefited from, fill in a new table for each.

Step 1:

Choose and describe an activity that run as part of the Pandemics course from which you believe you benefited:

………………………………………………………………………………………………
……………………

Immediate value	Potential value	Applied value	Realised value
Step 2:<br>Describe the change to you as a person as a result of this pandemics course-related activity	Step 3:<br>Describe a new idea, skill, relationship etc. that this 'pandemics' course-related activity generated for you and how you could change your practice	Step 4:<br>Describe the degree to which this insight, idea, skill affected your practice – what have it enabled you to do that would not have happened otherwise.<br>Rate (from 1 to 5 where 5 is to a large degree, 3 is moderate, and 1 is	Step 5:<br>Describe the degree to which this new practice has affected your success (impact of this new practice on your knowledge/skills relating to pandemic response in your context-<br>i.e. in your individual or organisational

		not at all)	context)<br><br>Rate (from 1 to 5 where 5 is too a large degree, 3 is moderate and 1 is not at all)
		Your rating:	Your rating:

References

E. Wenger, B.Trayner, and M.de Laat, "Promoting and assessing value creation in communities and networks: a conceptual framework". Rapport 18, Ruud de Moor Centrum, Open University of the Netherlands, 2011.
